# Non-Syndromic Hearing Loss in a Romanian Population: Carrier Status and Frequent Variants in the *GJB2* Gene

**DOI:** 10.3390/genes14010069

**Published:** 2022-12-26

**Authors:** Anca-Lelia Riza, Camelia Alkhzouz, Marius Farcaș, Andrei Pîrvu, Diana Miclea, Gheorghe Mihuț, Răzvan-Mihail Pleșea, Delia Ștefan, Mihaela Drodar, Călin Lazăr, on behalf of the HINT Study, on behalf of the FUSE Study, Mihai Ioana, Radu Popp

**Affiliations:** 1 Regional Centre of Medical Genetics Dolj, Emergency County Hospital Craiova, 200642 Craiova, Romania; 2 Laboratory of Human Genomics, University of Medicine and Pharmacy of Craiova, 200638 Craiova, Romania; 3 First Pediatric Department, “Iuliu Hatieganu” University of Medicine and Pharmacy, 400012 Cluj-Napoca, Romania; 4 Clinical Emergency Hospital for Children, 400394 Cluj-Napoca, Romania; 5 Molecular Sciences Department, “Iuliu Hatieganu” University of Medicine and Pharmacy, 400012 Cluj-Napoca, Romania; 6 ENT Department, Clinical Emergency Hospital for Children, 400394 Cluj-Napoca, Romania

**Keywords:** *GJB2* (gap-junction protein β 2), connexin 26, autosomal recessive non-syndromic hearing loss, deafness

## Abstract

The genetic causes of autosomal recessive nonsyndromic hearing loss (ARNSHL) are heterogeneous and highly ethnic-specific. We describe GJB2 (connexin 26) variants and carrier frequencies as part of our study and summarize previously reported ones for the Romanian population. In total, 284 unrelated children with bilateral congenital NSHL were enrolled between 2009 and 2018 in northwestern Romania. A tiered diagnostic approach was used: all subjects were tested for c.35delG, c.71G>A and deletions in *GJB6* (connexin 30) using PCR-based methods. Furthermore, 124 cases undiagnosed at this stage were analyzed by multiplex-ligation-dependent probe amplifications (MLPA), probe mix P163, and sequencing of *GJB2* exon 2. Targeted allele-specific PCR/restriction fragment length polymorphism (RFLP) established definite ethio-pathogenical diagnosis for 72/284 (25.35%) of the cohort. Out of the 124 further analyzed, in 12 cases (9.67%), we found compound heterozygous point mutations in *GJB2*. We identified one case of deletion of exon 1 of the *WFS1* (wolframin) gene. Carrier status evaluation used Illumina Infinium Global Screening Array (GSA) genotyping: the HINT cohort-416 individuals in northwest Romania, and the FUSE cohort-472 individuals in southwest Romania. GSA variants yielded a cumulated risk allele presence of 0.0284. A tiered diagnostic approach may be efficient in diagnosing ARNSHL. The summarized contributions to Romanian descriptive epidemiology of ARNSHL shows that pathogenic variants in the *GJB2* gene are frequent among NSHL cases and have high carrier rates, especially for c.35delG and c.71G>A. These findings may serve in health strategy development.

## 1. Introduction

Hearing impairment is a common sensory deficit with life-long personal and societal implications. Prevalence estimates have regional and age-specific differences. In 2018, hearing loss in children in central/east Europe had a prevalence of 1.5% [1] We could not identify reliable up-to-date estimates of hearing loss (HL) in Romania.

There are two main types of HL—sensorineural and conductive. Sensorineural hearing loss is the most common type; it impacts the inner ear and/or the pathways from the inner ear to the brain. Conductive HL is typically the result of obstructions in the outer or middle ear. A combination of sensorineural and conductive HL can also occur in mixed HL. Each type has different etiologies and prevalence reported, as well as different medical approaches to treatment.

Genetic causes account for approximatively half of the nonsyndromic sensorineural HL [2], in most cases with autosomal recessive nonsyndromic inheritance, leading to autosomal recessive nonsyndromic hearing loss (ARNSHL). More than 150 mendelian genes have been linked to hearing impairment, syndromic or non-syndromic [3]. More than 77 identified genes are involved in ARNSHL (hereditaryhearingloss.org accessed on 1 October 2022) [4,5].

The genetic determinants are however heterogeneous and highly ethnic-specific [6,7]. Common causative mutations found in *GJB2* (gap-junction protein β 2) and *GJB6* (gap-junction protein β 6) genes are reported in European, Middle Eastern [8], East Asian [9], Latino [10] and Jewish [11] populations, albeit with differences in frequencies for specific variants. The two genes are not as prevalently responsible for the etiology of hearing impairment in African descent [12,13], despite founder *GJB2* variants reported [14].

*GJB2* encodes connexin 26, which oligomerizes in a hexamer. Adjacent resulting connexons form a gap junction, critical for potassium homeostasis and cochlear development and maintenance [15,16]. Since described by Kelsell et al. [17], the DFNB locus including the *GJB2* gene has had more than 300 different pathogenic genetic variants identified as responsible for hearing impairment [18], with different mechanisms involved [19]. There are autosomal dominant non-syndromic or syndromic HL mutations in *GJB2* described [18].

In autosomal recessive transmission, a carrier inherits the variant allele from one parent and a normal allele from the other parent and therefore does not express the phenotype. Carrier frequencies have a gradient in European populations [20]. Reported mean carrier frequency is around 1.9% [20,21] with higher incidences toward the Mediterranean, estimated to 1 in 31 individual carriers in southern Europe [22]. Founder effect and hot spot may be involved in some variants in *GJB2* [23]. Overlap with migration patterns supports the founder hypothesis [24,25] for the most common *GJB2*:c.35delG in Europe and the Middle East and questions its evolutionary significance. The *GJB2*:c.71G>A (p.W24X) variant is frequent in Ashkenazi Jews [11].

Phenotype correlations for *GJB2* pathogenic variants are not straightforward [26]. The audiological features vary greatly, but there seems to be a gradient from more severe profound bi-lateral hearing loss in the case of homozygous truncating mutation c.35delG to milder hearing phenotypes in missense mutations [7,18,27]. ARNSHL is in most cases prelingual, mostly congenital and stable [19], although progression of hearing loss is surprisingly common [28]. 

Connexin 30, encoded by *GJB6,* also causes moderate to profound hereditary hearing loss. Within the over 20 pathogenic variants reported leading to ARNSHL, it is especially the large deletions in the *GJB2* or *GJB6* genes that lead to hearing impairment either in a homozygous, heterozygous or compound heterozygous state [18,29].

Sensorineural HL patients can benefit from medical treatment. Cochlear implantation (CI) is the most important and effective approach for profound sensorineural HL. Most authors concur that patients with genetic causes involving an ‘intra-cochlear’ etiology, *GJB2* included, show good outcomes after CI. [30,31]

The aims of the current study were:

(1) Describe the *GJB2* gene pathogenic variant frequencies in a population of hearing-impaired children in northwestern Romania and compare these with other genetic findings in similar Romanian cohorts;
(2) Report on the carrier status for more frequent variants in *GJB2* in two Romanian cohorts to contribute to current knowledge needed for genetic diagnosis, counseling and strategy making for genetic screening and diagnosis of deafness.

## 2. Materials and Methods

### 2.1. Diagnostic Group

The study protocol was approved by the Ethics Board of the University of Cluj-Napoca, approval no. 25/2009. For all patients undergoing audiological and genetic evaluation, written consent was obtained according to the World Medical Association Declaration of Helsinki.

#### 2.1.1. Patient Inclusion

The study group consists in part of a previously established cohort of patients from 10 different counties in northwestern Romania examined in the Pediatric Department of the Pediatric Hospital Cluj. Initial enrollment started in 2009 [32], and continued until 2018, allowing us to include 284 unrelated children aged <18 years, with bilateral congenital nonsyndromic sensorineural hearing loss with prelingual onset. Male:female ratio was 1:1; mean age was 11.18 ± 6.39. Hearing loss cases secondary to other recognized factors related to the pregnancy period, birth and neonatal events, unilateral HL ototoxic treatments, infections, tumors, etc. [33,34], were excluded from the study, as were syndromic cases. The declared ethnicity of the subjects was Romanian.

Patient work-up was previously described [32]; it included clinical and paraclinical examination, complete ENT examination with audiological examinations, and thorough personal and familial history.

#### 2.1.2. Molecular Testing

DNA extraction was performed from EDTA (ethylenediaminetetraacetic acid) peripheral venous blood using a commercially available kit (Wizard Genomic DNA Purification Kit, Promega, Madison, WI, USA).

Targeted techniques and MLPA (Multiplex Ligation-dependent Probe Amplification) for identification of ARNSHL frequent pathogenic variants were performed at the Dept. of Genetics, University of Medicine and Pharmacy of Cluj. Sequencing was performed at the Laboratory of Medical Genetics, Regional Centre of Medical Genetics Dolj. 

All 284 subjects enrolled underwent targeted testing for a selection of pathogenic variants. Out of these, 124 cases undiagnosed by the initial testing went ahead to MLPA and sequencing of the *GJB2* gene. In total, 88 undiagnosed cases did not proceed because they did not consent to a second test or a second blood drawn being performed or could not be contacted further.

#### 2.1.3. Semi-Nested PCR-RFLP and ARMS-PCR

The PCR-based methods included detection of two common *GJB2* variants: c.35delG using semi-nested PCR technique followed by RFLP as well as ARMS-PCR analysis; and c.71G>A by ARMS-PCR analysis [17,32,35,36].

#### 2.1.4. MLPA

MRC-Holland probe mix P163 GJB-WFS1-POU3F4 was used according to the manufacturer’s protocol to evaluate micro-deletions or duplications in a selection of genes: *GJB2*, *GJB3*, *GJB6*, POU3F4; and targeted specific common variants in the *GJB2* gene: c.35delG, c.101T>C, c.167delT, c.235delC, and c.313_326del14. Results generated on ABI 3500 Genetic Analyzer using a 36 cm array and POP7 polymer (Applied Biosystems, Waltham, MA, USA) were analyzed using Coffalyser.NET (MRC-Holland, Amsterdam, The Netherlands).

#### 2.1.5. Sanger Sequencing by Capillary Electrophoresis

The open reading frame of coding exon 2 of the *GJB2* gene was amplified using primers and PCR conditions previously described [37]. Applied Biosystems™ BigDye™ Terminator v1.1 Cycle Sequencing Kit and clean-up (Thermo Fisher, Waltham, MA, USA) was used. Sequencing was performed on an ABI 3730 Genetic Analyzer—36 cm array and POP7 polymer (Applied Biosystems). Data analysis was performed using Mutation Surveyor^®^ DNA Variant Analysis Software v.5 (Softgenetics, State College, PA, USA). Variant classification followed American College of Medical Genetics and Genomics (ACMG) recommendations and consulted online databases ClinVar, Varsome [38]. The guidance ACMG developed on the interpretation of variants identified in Mendelian disorders recommends evidence-based classification of variants into five categories: ‘pathogenic’, ‘likely pathogenic’, ‘uncertain significance’, ‘likely benign’, and ‘benign’ [39]. Deafness Variation Database was consulted for pathogenicity calls at https://deafnessvariationdatabase.org (accessed on 16 October 2022) [40].

### 2.2. Carrier Status Evaluation

For carrier status evaluation, we relied on array-based genotyping performed on Romanian population (unpublished data), which we interrogated anonymously, at population level. 

Healthy unrelated Romanians were enrolled in two cohorts, as part of two research projects including genotyping: (1) the HINT cohort, northwest Romania, 416 individuals, and (2) the FUSE cohort, southwest regions of Romania, 472 individuals. Age range was 18-98 years old, with roughly equal male:female ratio.

The HINT study was approved by the Research Ethics Committee of Iuliu Haţieganu University of Medicine and Pharmacy, Cluj-Napoca (425/24 November 2016).

The FUSE study protocol was approved by the Committee of Ethics and Academic and Scientific Deontology from the University of Medicine and Pharmacy of Craiova (80/17 November 2016). All participants signed an informed consent form.

#### Genotyping Data

Genotyping was performed using Infinium Global Screening Array (GSA)-24 BeadChip on illumina iScan platforms. GSA v1.1 was used for the Cluj cohort, with genotyping performed at Rotterdam University Medical Centre, the Netherlands, and GSA v3.0 for the Craiova cohort, genotyping performed as part of the collaboration with the Genetics Department University Medical Centre Groningen, the Netherlands.

For the current study, we were restricted to variants passing quality filters that were present on the chip between the *GJB2* coordinates (GRCh37) chr13:20,761,609 and chr13:20,767,077. Data analysis was performed using Illumina Genome Studio v2.0 (Illumina, San Diego, CA, USA).

Allele frequencies for the variants of interest were checked in 1000G EUR (European population using Variant Effect Predictor—online interface) [41].

## 3. Results

### 3.1. Diagnostic Group

**Figure 1:**
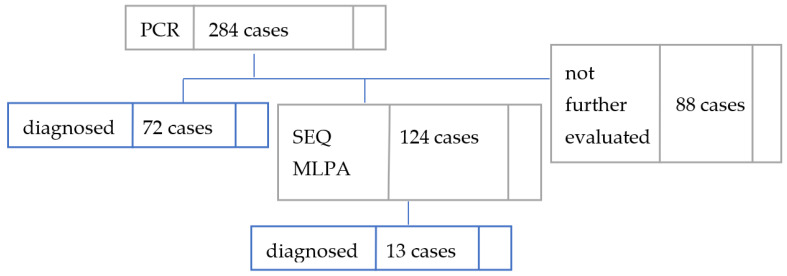
Diagnosis workflow in our study: 284 cases underwent targeted testing; 124 of them accepted to have sequencing of *GJB2* exon 2 and MLPA-P163 testing performed. PCR—polymerase chain reaction, SEQ—sequencing, MLPA—multiplex-ligation-dependent probe amplification.

Our study included 284 unrelated children with bilateral congenital severe to profound NSHL. A tiered molecular diagnosis approach was taken, as Figure 1 below illustrates.

PCR-based techniques were used as a first-tier approach to evaluate the presence of c.35delG, c.71G>A and *GJB6* variants; results are shown in Table 1. Targeted techniques were able to establish definite ethio-pathogenical diagnosis for 72/284 (25.35%) of the cohort, as either homozygous c.35delG (62 cases, 86.11% of the positive cases), homozygous c.71G>A (5 cases, 6.94% of the positive cases), compound heterozygous c.35delG/c.71G>A (4 cases, 5.55%), or compound heterozygous c.35delG/del *GJB6*-D13S183 (1 case, 1.38%). An additional 30/284 (10.56%) patients carried one allele c.35delG. We identified four carriers of c.71G>A following this initial assessment.

In total, 124 cases, 17 of which were c.35delG heterozygous, were further analyzed by both MLPA and sequencing as part of the second-tier diagnostic approach; results are shown in Table 2. 

MLPA detected one individual with deletion of exon 1 of the gene WFS1.

Out of the 124, in 12 cases (9.67%), sequencing identified compound heterozygous of point mutations in *GJB2*. Ten of these were compound heterozygous of c.35delG/c.551G>C in five cases; c.35delG/c.269T>C, c.35delG/c.299_300delAT, c.35delG/c.101T>G, c.35delG/c.370C>T in one case each; in one case, we found a compound genotype c.35delG/c.314_329del. We also identified compound heterozygous case c.71G>A/c.551G>C. Another complex diagnosis was c.299_300delAT/c.314_329del.

In our setup, the use of a tiered diagnostic approach and lack of inclusion of all individuals after the PCR step does not allow for a true comparison between the diagnostic methods in use. However, if we are to estimate diagnosis success rates:

- targeted PCR for two common variants (c.35delG, c.71G>A) would be 25.35% (72/284);
- MLPA P163 would be at best 33.67% (66/196);
- sequencing for exon 2 of the GJB2 gene could reach up to 42.85% (84/196);
- MLPA P163 in conjunction with sequencing for exon 2 of the GJB2 gene could reach up to 43.36% (85/196).

### 3.2. Carrier Status Evaluation

GSA v1 and GSA v3 genotyping chips combine multi-ethnic genome-wide content on its ~654,027 fixed markers. We are reporting the carrier status of those variants in the *GJB2* gene, with MAF>0 in two cohorts of unrelated, healthy Romanians (see Table 3) from Cluj and Craiova. Supplementary Table S1 includes all 55 and 63 variants present on the two chips. 

## 4. Discussion

### 4.1. Diagnosis Findings in Context

Several studies conducted in Romania have either evaluated common mutations reported in European populations or sequenced this gene to unravel the genetic causes in this population. 

Contributions to Romanian descriptive epidemiology of ARNSHL frequent variants, including detailed results of the current study, are summarized in Table 4. Inclusion criteria for the studies were bilateral hearing impairment throughout the selected studies, whether from mild to profound [42,43], or from severe to profound [44,45]. Radulescu et al. [44] focused on CI patients. All studies excluded syndromic or environmental/infectious etiology for hearing loss. Additionally, we restricted the variants listed to pathogenic or likely pathogenic, following ACMG scoring recommendations. Table layout allows for an overview on allele zygosity state.

As Table 4 shows, c.35delG was the most frequent *GJB2* pathogenic variant identified in Romanian studies, followed at a relatively large difference by c.71G>A. Lazăr et al. [32] reported an allele frequency of 33.3% for c.35delG and 5.3% for c.71G>A in their deafness study group. These initial findings of part of our cohort are backed by the current study frequencies of c.35delG and c.71G>A—in the current cohort, allele frequency for c.35delG is 27.99% and c.71G>A of 3.16%. Taking this into context of the literature summary that Table 4 offers, it is safe to conclude that these two-point mutations in the *GJB2* gene alone cover most of the genetic profile; this supports their use as main variants for targeted ARNSHL testing in the Romanian population.

Overall, for 10/17 cases carrying c.35delG, we could identify a second mutation in the *GJB2* gene. The added value of sequencing the *GJB2* gene is therefore especially relevant for this subgroup, given that only 2/124 additional cases were compound heterozygous of different variants than the two most frequent.

Conversely, this means that 7/17 cases were monoallelic. To speculate, this can be the result of testing limitations. Despite its extended scope of analysis, WES studies also report heterozygous *GJB2* mutations in HL cases [46]. Although in rare cases, uniparental disomy can occur to explain the phenotype, rarely, a second disease-causing pathogenic variant in the DNB1 locus is found; exome sequencing mostly identifies the involvement of other genes [47]. Additionally, calling pathogenicity is challenging; variant reclassification can occur, and therefore, diagnoses may need to be revisited.

The configuration of pathogenic variants differs slightly between studies. Resmeriță et al. [42] also discussed the apparent lack of homozygous c.71G>A and considered it a sampling issue in the context of an ethnicity-driven condition. Our study reports several pathogenic/likely pathogenic variants for the first time in the Romanian population, and we find quite unique cases to be the multiple-variant compound heterozygous cases.

The spectrum of *GJB2* variation is known to be ethnic-specific [24,25]. We did not find in any of the Romanian studies identified information on the region of Romania where the enrolled patients originate or their declared ethnicity. Nonetheless, although the picture is still unclear, there seem to not be significant regional differences; future larger studies may shed light on this further. 

Declared ethnicity may not always reflect descendance. This is of relevance in the context of nation-wide plans for genetic testing, which should be adjusted to the population characteristics. For instance, referring to Romania’s minorities, Hungarian studies report a slightly lower implication of the *GJB2* gene [48]. A high frequency of *GJB2* mutation c.71G>A is reported in the Rroma, as well as in the Indian population [49,50]. Our study identifies c.71G>A as the second common pathogenic variant. Resmeriță et al. [42] noted the lower than expected rate of discovery for c.71G>A in their study and justly proposed that separate studies are needed for the Rroma. To speculate, small differences between rates seen in Table 4 may be caused by sample size, enrollment criteria, ethnicity recording, patient pool tested, addressability for medical services of different ethnicities as well. 

All studies that looked at previously reported micro-deletion and duplications concur that they are rare in the Romanians. Our study identifies one del WFS1 exon 1/126 patients tested [42], finds four cases of del WFS 1–8 and three cases of del *GJB2* exon 1/291 subjects evaluated, [44] and does not identify any case with del(*GJB6*-D13S1830), del(*GJB6*-D13S1854) and del (chr13: 19,837,343–19,968,698). *GJB6* has been proven to be otherwise rare in central Europe [29].

### 4.2. Carrier Findings in Context

Based on southwestern Romania data, our study sets the carrier rate for c.35delG at 3.81%, and a possible carrier rate of pathogenic or likely pathogenic variants of at least 5.72%. This carrier rate is comparable to the previously described 3.14%, reported by evaluating 350 unrelated fetal DNA samples [51], a carrier rate of c.35delG otherwise comparable to most southeastern European populations [22]. 

By comparing the overlapping covered variants between the two Romanian genotyping datasets in Table 3, we do not identify major differences. Nonetheless, there are discrepancies in the public data on the European population, covered more extensively in Supplementary Table S1. This is yet another argument that ethnicity-based epidemiological data on pathogenic variants and carrier frequency are essential to inform clinical and political decision making. 

The high prevalence of c.35delG in the HL diagnostic cohorts and the high carrier rate in the population for pathogenic alleles are compelling arguments to make it a prime candidate for genetic screening. 

### 4.3. Methods and Approaches for Testing GJB2

Since the 2000s, the list of techniques seen as appropriate for genetic testing for c.35delG included allele-specific PCR assays or other, more complex techniques such as single-strand conformational polymorphism analysis, denaturing high-performance liquid chromatography (DHPLC) or heteroduplex analysis. DNA sequencing can also be used; it is seen as the golden standard against which all other screening methods must be compared [52], or as a second-step technique to search for additional alleles [53]. Recent large studies have also used real-time PCR [54,55] or microarray [56].

In ARNSHL, targeted PCR for the most frequent pathogenic variants has a chance to reach rather high diagnostic rates. In our study, PCR-based techniques for two of the most frequent mutations were able to establish definite ethio-pathogenical diagnosis for 25.35% and bring a partial result for an additional 11.97% of individuals tested. 

Combinations of PCR and sequencing applied in a tiered fashion have been used before [57]. In terms of cost, and perhaps time to result, the approach seems to have its benefits. Our own diagnostic rate estimates in Table 2 situate sequencing of exon 2 of the *GJB2* gene between 30 and 40%, and with modest additional benefits, MLPA P163 should also be used. Our findings are comparable to other Romanian reported rates and seem to suggest a comparable rate should a PCR-based approach for c.35delG and c.71G>A or MLPA P163 be used. When sequencing is not possible, both of these techniques would be cost-effective pragmatic options. An equivalent of the PCR techniques could also be RT-PCR, and Sanger sequencing could be swapped with targeted next-generation sequencing (NGS) techniques [58].

The introduction of NGS has resulted in great progress in diagnostics, allowing for one to study all known HL genes in a single assay. The diagnostic yield is currently still less than 60% [59] but has the potential to increase substantially. There is certainly a role for exome sequencing in hearing loss diagnosis [12,59,60,61,62,63,64]. All these show the unavoidable trend for genomic sequencing to become standard of care [65]. NGS panels and exome sequencing can detect most pathogenic sequence and copy number variants that cause NSHL; however, additional assays to capture all pathogenic copy number variants are required. Adoption of genome sequencing may simplify diagnostic workflows, but further investigational studies will be required to evaluate its clinical efficacy [66]. Not to be overlooked are the implications and challenges for counseling [65]. 

The main challenge for the future will be establishing population-specific variation spectra to achieve accurate personalized molecular testing [67]. Based on current literature reports, *GJB2* testing remains an efficient first-tier gesture in evaluating an infant with unexplained congenital hearing loss. Second-tier testing can include panels or whole exome sequencing strategies and are becoming widely used in clinical practice worldwide. Referral to medical genetics should always be considered in cases without identified causes for hearing loss and in recurrence risk assessment [68].

## 5. Conclusions

We have reported *GJB2* and *GJB6* pathogenic variants in a population of hearing-impaired children in northwestern Romania and summarized frequent pathogenic variants reported in similar Romanian cohorts. We showed the high contribution of c.35delG, followed by c.71G>A, c.551G>C, c.101T>C and other point mutations in *GJB2*, with slight apparent regional differences. 

We showed that ARMS-PCR/RFLP can diagnose one-fourth of the NSHL cases. Capillary sequencing could reach close to a 40% diagnostic rate. A tiered approach can be an option in resource-limited settings.

Carrier status of variants qualified as likely pathogenic or pathogenic present on the GSA places the Romanian population at a cumulated risk allele presence of 0.0284, comparable to most southeastern European populations.

Reported frequencies can be helpful for genetic diagnosis, counseling and ultimately strategy making for genetic screening and diagnosis of deafness.

## Figures and Tables

**Table 1:** Diagnosis results for using targeted PCR for common variants as first-tier diagnostic means.

	Targeted PCR for Common Variants *GJB2* (c.35delG, c.71G>A) and Large Deletions on *GJB6*
**Subjects evaluated (*n*)**	**284**
**Definite diagnosis**	**72**
c.35delG/c.35delG	62
c.71G>A/c.71G>A c.35delG/c.71G>A c.35delG/del GJB6-D13S183	5 4 1
**Monoallelic cases**	**34**
c.35delG/? c.71G>A/?	30 4
**No diagnosis**	**178**

**Table 2:** Diagnosis results for using MLPA P163 and sequencing of open reading frame of exon 2 *GJB2* gene as second-tier diagnostic means, following targeted PCR for common variants.

	MLPA P163 and Sequencing Exon 2 *GJB2*
**Subjects evaluated (*n*)**	**124**
**Definite diagnosis**	**12**
c.35delG/c.551G>C c.35delG/c.101T>G c.35delG/c.269T>C c.35delG/c.370C>T c.35delG/c.314_329del c.35delG/exon 1 WFS1 c.71G>A/c.551G>C c.299_300delAT/c.314_329del	5 1 1 1 1 1 1 1
Monoallelic cases *	**9**
c.35delG/? c.71G>A/?	7 2
**No diagnosis**	**103**

* reported only for the two frequent mutations.

**Table 3:** Carrier status of variants qualified as likely pathogenic or pathogenic present on the GSA v1 or GSA v3 chips, with at least one allele identified in the Romanian cohorts evaluated.

Chr:Pos (GRCh37)	Identifier Rs NM	Nucleotide Change (DNA)	ClinVar	GSA v1 Cluj *n* = 416 (AF)	GSA v3 Craiova *n* = 472 (AF)	AF 1000 G EUR
**13:20763395**	rs111033253 NM_004004.6: c.313_326del	CCCTT GATGA ACTTC>C	Pathogenic	0	1/472 (0.0010)	-
**13:20763452**	rs80338945 NM_004004.6: c.269T>C (p.Leu90Pro)	A>G	Pathogenic	NA	1/472 (0.0010)	-
**13:20763612**	rs72474224 NM_004004.6(*GJB2*): c.109G>T (p.Val37Phe) NM_004004.6(*GJB2*): c.109G>A (p.Val37Ile)	C>A C>T	Likely pathogenic Pathogenic	0 3/416 (0.0036)	0 1/472 (0.0010)	- 0
**13:20763620**	rs35887622 NM_004004.6(*GJB2*): c.101T>G (p.Met34Arg) NM_004004.6(*GJB2*): c.101T>C (p.Met34Thr)	A>C A>G	Likely pathogenic Pathogenic	0 5/416 (0.0060)	0 5/472 (0.0053)	- 0.0209
**13:20763686**	rs80338939 NM_004004.6(*GJB2*): c.35del (p.Gly12fs) NM_004004.6(*GJB2*): c.35dup (p.Val13fs)	CC>C CC>CCC	Pathogenic Uncertain	NA NA	18/472 (0.0191) 0	0.0089 -
**13:20766921**	rs80338940 NM_004004.6: c.-23 + 1G>A	C>T	Pathogenic	0	1/472 (0.0010)	-
**Cumulated risk allele presence**					27/472 (0.0284)	

AF—allele frequency, NA – not evaluated on the GSA chip, 1000 Genomes, EUR—European.

**Table 4:** Pathogenic or likely pathogenic variants described in our cohort as well as other Romanian cohorts published to date. Deafness variation database was consulted and pathogenicity calls coincided, where available.

Identifier Rs (dbSNP 154) *GJB2*-NM_004004.6 Protein Change	ACMG Score	Current Study	Resmeriță et al. [42]	Rădulescu et al. [44]	Dragomir et al. [43]	Neagu et al. [45]
Region in Romania *		**Northwestern**	**Northeastern**	**Eastern**	**Southern**	**Southern**
rs80338940 c.-23 + 1G>A	Pathogenic (PP5, PVS1, PM2)	-	6 Het	-	3 Hom	-
rs80338939 c.35delG p.G12Vfs * 2	Pathogenic (PS3, PVS1, PP5)	62 Hom 15 C/H 20 Het	57 Hom 30 C/H 26 Het	12 Hom 5 C/H 3 Het	46 Hom 6 C/H 5 Het	10 Hom 2 C/H
rs104894396 c.71G>A p.Trp24Ter	Pathogenic (PP5, PVS1, PM2)	5 Hom 5 C/H 3 Het	8 C/H 7 Het	2 C/H	3 Hom 6 C/H 2 Het	2 Hom 2 C/H 1 Het
rs564084861 c.100A>T p.Met34Leu	Pathogenic (PM1, PM5, PM2, PP2)	-	3 Het	-	-	NA
rs35887622 c.101T>C p.Met34Thr	Pathogenic (PS3, PM1, PM5, PP5, PP2)	1 C/H 2 Het	10 C/H 9 Het	-	-	NA
rs72474224 c.109G>A p.Val37Ile	Pathogenic (PP5, PM1, PM5, PS1, PP2)	-	3 Het	-	-	NA
rs80338945 c.269T>C p.Leu90Pro	Pathogenic (PP5, PM1, PM2, PP2, PP3)	1 C/H 1 Het	3 Het	-	-	NA
rs111033204 c.299_300delAT p.His100ArgfsTer14	Pathogenic (PP5, PVS1, PM2)	1 C/H		1 C/H	-	NA
rs111033253 c.313_326del p.Lys105GlyfsTer5	Pathogenic (PVS1, PP5, PM2)	-	6 C/H 2 Het	2 C/H	-	NA
rs797045596 c.314_329del p.Lys105ArgfsTer2	Pathogenic (PVS1, PM2, PP5)	1 C/H		-	-	NA
rs80338947 c.358_360delGAG p.Glu120del	Pathogenic (PP5, PM1, PM2, PM4, PP3)	-	-	1 Het	-	NA
rs397516874 c.370C>T p.Gln124Ter	Pathogenic (PVS1, PP5, PM2)	1 C/H	-	-	-	NA
rs80338950 c.551G>C p.Arg184Pro	Pathogenic (PM1, PM2, PM5, PP5, PP2, PP3)	6 C/H 1 Het	2 C/H 2 Het	2 C/H	-	NA
in-del		1 del WFS1 exon 1	3 del *GJB2* exon 1 4 del WFS exons 1-8	NA	NA	NA
Cases included, testing strategy		***n* = 284** ** targeted c.35delG, c.71G>A; out of which ***n* = 126** followed with capillary sequencing and MLPA	***n* = 291** capillary sequencing and MLPA	***n* = 45** capillary sequencing	***n* = 125** targeted c.35delG; out of which ***n* = 79** capillary sequencing	***n* = 34** targeted c.35delG c.71G>A
Definite etio-pathogenic diagnosis **		* 72/284 (25.35%)	92/291 (31.61%)	18/45 (40%)	52/125 (44%)	12/34 (41.17%)
Monoallelic cases ***		* 34/284 (11.97%)	61/291 (20.96%)	4/45 (8.89%)	7/125 (5.6%)	1/34 (2.94%)

* reported ethnicity judgement based on affiliations and methods section information provided by the authors. ** diagnosis rate is calculated in the table for the targeted approach only. *** monoallelic cases—only one affected allele detected. Hom—homozygous; C/H—compound heterozygous; Het—heterozygous.

## Data Availability

Not applicable.

## Supplementary Materials

The following supporting information can be downloaded at: https://www.mdpi.com/article/10.3390/genes14010069/s1, Table S1: Carrier status of variants qualified as likely pathogenic or pathogenic present on the GSA v1 or GSA v3 chips identified in the Romanian cohorts evaluated.
