# Supplementary material for: Non-Syndromic Hearing Loss in a Romanian Population: Carrier Status and Frequent Variants in the GJB2 Gene
Source: Genes (Basel). 2022 Dec 26;14(1):69. doi: 10.3390/genes14010069 (PMC9858611; doi:10.3390/genes14010069)
Supplement: Supplementary file 1 [file genes-14-00069-s001.zip › genes-2004703-supplementary.pdf]

genes

MDPI

Supplementary Table S1. Carrier status of variants qualified as likely pathogenic or pathogenic present on the GSA v1 or v3 chips identified in GSA the Romanian cohorts evaluated. The reported numbers reflect heterozygous individuals/allele counts.

**Chr:Pos (GRCh37)**	**Identifier rs (dbSNPv154) NM (GJB2)**	**ClinVar**	**GSAv1 Cluj *n*=416**	**GSAv3 Craiova *n*=472**	**AF 1000G EUR**
13:20763039	rs111033327<br>NM_004004.6:c.*1C>T	Uncertain significance	NA	0	0.001
13:20763071	rs587783647<br>NM_004004.6:c.647_650del<br>p.Arg216fs	Pathogenic/Likely pathogenic	0	0	-
13:20763088	rs587783646<br>NM_004004.6:c.632_633del<br>NM_004004.6:c.632_633delGT<br>p.Cys211fs	Pathogenic/Likely pathogenic	0	0	-
13:20763104	rs111033294<br>NM_004004.6:c.617A>G<br>p.Asn206Ser	Pathogenic/Likely pathogenic	0	0	-
13:20763113	rs76838169<br>NM_004004.6:c.608T>C<br>p.Ile203Thr	Benign	0	0	0
13:20763121	rs111033335<br>NM_004004.6:c.592_600<br>delinsCAGTGTTCATGACATTC<br>p.Val198_Gly200delinsGlnCysSerTer	Pathogenic/Likely pathogenic	0	0	-
13:20763123	rs786204597<br>NM_004004.6:c.598G>A<br>p.Gly200Arg	Pathogenic/Likely pathogenic	NA	0	-
	NM_004004.6:c.598G>T<br>p.Gly200Ter	Likely pathogenic	NA	0	-
13:20763170	rs80338950<br>NM_004004.6:c.551G>A<br>p.Arg184Gln	Pathogenic/Likely pathogenic	0	0	-
	NM_004004.6:c.551G>C<br>p.Arg184Pro	Pathogenic	0	0	-
13:20763186	rs28931595				

	NM_004004.6:c.535G>C p.Asp179His	Uncertain significance	0	0	-
	NM_004004.6:c.535G>A p.Asp179Asn	Pathogenic	0	0	-
13:20763222	rs111033360 NM_004004.6:c.499G>A p.Val167Met	Uncertain significance	0	0	0
13:20763228	rs376898963 NM_004004.6:c.493C>T p.Arg165Trp	Uncertain significance	NA	0	0
13:20763234	rs80338949 NM_004004.6:c.487A>C p.Met163Leu	Uncertain significance	0	0	-
	NM_004004.6:c.487A>G p.Met163Val	Uncertain significance	2/416	0	0
13:20763243	rs34988750 NM_004004.6:c.478G>A p.Gly160Ser	Uncertain significance	1/416	1/472	0
13:20763256	rs772264564 NM_004004.6:c.465T>A p.Tyr155Ter	Pathogenic	0	0	-
13:20763265	rs111033420 NM_004004.6:c.456C>T p.Tyr152=	Likely benign	0	0	-
	NM_004004.6:c.456C>A p.Tyr152Ter	Pathogenic/Likely pathogenic	0	0	-
13:20763293	rs104894401 NM_004004.6:c.428G>A p.Arg143Gln	Pathogenic/Likely pathogenic	NA	0	-
13:20763294	rs80338948 NM_004004.6:c.427C>T p.Arg143Trp	Pathogenic	0	0	0
13:20763295	rs397516877 NM_004004.6:c.426C>A p.Phe142Leu	Pathogenic	0	0	-
13:20763296	rs116769964 NM_004004.6:c.425T>C p.Phe142Ser	Uncertain significance	0	0	0
13:20763305	rs76434661				

	NM_004004.6:c.416G>A<br>p.Ser139Asn	Pathogenic	0	0	0.001
13:20763313	rs786204690<br>NM_004004.6:c.408C>A<br>p.Tyr136Ter	Pathogenic/Likely pathogenic	NA	0	-
	NM_004004.5:c.[134G>A;408C>A]	Pathogenic	NA	0	
13:20763332	rs779018464<br>NM_004004.6:c.389G>C<br>p.Gly130Ala	Likely pathogenic	NA	0	-
13:20763351	rs397516874<br>NM_004004.6:c.370C>T<br>p.Gln124Ter	Pathogenic	0	0	0
13:20763356	rs111033295<br>NM_004004.6:c.365A>T<br>p.Lys122Ile	Pathogenic/Likely pathogenic	0	0	-
13:20763361	rs80338947<br>NM_004004.6:c.355GAG<br>p.Glu120del	Pathogenic	0	0	-
13:20763366	rs150529554<br>NM_004004.6:c.355G>T<br>p.Glu119Ter	Pathogenic/Likely pathogenic	NA	0	-
	NM_004004.6:c.355G>A<br>p.Glu119Lys	Uncertain significance	NA	0	0.001
13:20763371	rs111033441<br>NM_004004.6:c.350A>G<br>p.Asp117Gly	Uncertain significance	0	0	-
13:20763380	rs2274083<br>NM_004004.6:c.341A>G<br>p.Glu114Gly	Benign	NA	0	0
	NM_004004.5:c.[79G>A;341A>G]	Benign/Likely benign	NA	0	
13:20763382	rs80338946<br>NM_004004.6:c.339T>G<br>p.Ser113Arg	Uncertain significance	NA	0	-
13:20763394	rs797045596<br>NM_004004.6:c.314_329del<br>p.Lys105fs	Pathogenic	0	0	-
**13:20763395**	**rs111033253**<br>**NM_004004.6:c.313_326del**<br>**p.Lys105fs**	**Pathogenic/Likely pathogenic**	0	1/472	-

13:20763421	rs111033204<br>NM_004004.6:c.299_300del<br>p.His100fs	Pathogenic	0	0	-
13:20763423	rs143343083<br>NM_004004.6:c.298C>T<br>p.His100Tyr	Pathogenic/Likely pathogenic	0	0	-
13:20763438	rs111033299<br>NM_004004.6:c.283G>A<br>p.Val95Met	Pathogenic/Likely pathogenic	0	0	0
**13:20763452**	**rs80338945**<br>**NM_004004.6:c.269T>C**<br>**p.Leu90Pro**	**Pathogenic**	NA	1/472	-
13:20763471	rs104894409<br>NM_004004.6:c.250G>T<br>p.Val84Leu	Pathogenic	NA	0	-
	NM_004004.6:c.250G>A<br>p.Val84Met	Pathogenic	NA	0	-
	NM_004004.6:c.250G>C<br>p.Val84Leu	Pathogenic	NA	0	-
13:20763482	rs727504302<br>NM_004004.6:c.239A>C<br>p.Gln80Pro	Pathogenic/Likely pathogenic	0	0	-
13:20763486	rs80338943<br>NM_004004.6:c.235del<br>p.Leu79fs	Pathogenic	0	0	0
13:20763490	rs80338944<br>NM_004004.6:c.231G>A<br>p.Trp77Ter	Pathogenic	0	0	0
13:20763491	rs104894395<br>NM_004004.6:c.230G>A<br>p.Trp77Ter	Pathogenic/Likely pathogenic	NA	0	-
13:20763492	rs104894397<br>NM_004004.6:c.229T>C<br>p.Trp77Arg	Pathogenic	NA	0	-
13:20763494	rs111033361<br>NM_004004.6:c.227T>C<br>p.Leu76Pro	Likely pathogenic	0	0	-
13:20763497	rs28931593<br>NM_004004.6:c.224G>A	Pathogenic	0	0	-

	p.Arg75Gln				
13:20763498	rs104894402				
	NM_004004.6:c.223C>G p.Arg75Gly	Likely pathogenic	0	0	-
	NM_004004.6:c.223C>T p.Arg75Trp	Pathogenic	0	0	-
13:20763545	rs104894404				
	NM_004004.6:c.176G>A p.Gly59Asp	Likely pathogenic	0	0	-
	NM_004004.6:c.176G>C p.Gly59Ala	Pathogenic	0	0	-
13:20763552	rs111033297				
	NM_004004.6:c.169C>T p.Gln57Ter	Pathogenic	0	0	-
13:20763554	rs80338942				
	NM_004004.6:c.167del p.Leu56fs	Pathogenic	NA	0	-
13:20763563	rs587783645				
	NM_004004.6:c.158G>T p.Cys53Phe	Likely pathogenic	0	0	-
	NM_004004.6:c.158G>A p.Cys53Tyr	Pathogenic	0	0	-
13:20763573	rs28931594				
	NM_004004.6:c.148G>T p.Asp50Tyr	Pathogenic	0	0	-
	NM_004004.6:c.148G>A p.Asp50Asn	Pathogenic	0	0	-
13:20763582	rs104894398				
	NM_004004.6:c.139G>T p.Glu47Ter	Pathogenic	0	0	-
13:20763587	rs72561723				
	NM_004004.5:c.[134G>A;408C>A]	Pathogenic	0	0	-
	NM_004004.6:c.134G>A p.Gly45Glu	Pathogenic/Likely pathogenic	0	0	
13:20763589	rs104894407				
	NM_004004.6:c.132G>A p.Trp44Ter	Pathogenic	0	0	0
	NM_004004.6:c.132G>C p.Trp44Cys	Pathogenic	0	0	-

13:20763590	rs104894413				
	NM_004004.6:c.131G>A<br>p.Trp44Ter	Pathogenic/Likely pathogenic	0	0	-
	NM_004004.6:c.131G>C<br>p.Trp44Ser	Pathogenic	0	0	-
13:20763601	rs561870637				
	NM_004004.6:c.120A>C<br>p.Ala40=	Likely benign	NA	0	0.003
13:20763602	rs111033296				
	NM_004004.6:c.119C>G<br>p.Ala40Gly	Likely pathogenic	0	0	-
	NM_004004.6:c.119C>A<br>p.Ala40Glu	Likely pathogenic	0	0	-
**13:20763612**	**rs72474224**				
	**NM_004004.6:c.109G>T**<br>**p.Val37Phe**	**Likely pathogenic**	0	0	-
	**NM_004004.6:c.109G>A**<br>**p.Val37Ile**	**Pathogenic**	3/416	1/472	0
13:20763614	rs587783644				
	NM_004004.6:c.107T>C<br>p.Leu36Pro	Uncertain significance	0	0	-
**13:20763620**	**rs35887622**				
	**NM_004004.6:c.101T>G**<br>**p.Met34Arg**	**Likely pathogenic**	0	0	-
	**NM_004004.6:c.101T>C**<br>**p.Met34Thr**	**Pathogenic**	5/416	5/472	0.0209
13:20763626	rs111033190				
	NM_004004.6:c.95G>T<br>p.Arg32Leu	Pathogenic/Likely pathogenic	0	0	-
	NM_004004.6:c.95G>A<br>p.Arg32His	Pathogenic	0	0	-
13:20763627	rs371024165				
	NM_004004.6:c.94C>A<br>p.Arg32Ser	Pathogenic/Likely pathogenic	NA	0	-
	NM_004004.6:c.94C>T<br>p.Arg32Cys	Pathogenic	NA	0	-
13:20763642	rs2274084				
	NM_004004.5:c.[79G>A;341A>G]	Benign/Likely benign	0	0	0
	NM_004004.6:c.79G>A	Benign	4/416	7/472	

	p.Val27Ile				
13:20763650	rs104894396<br>NM_004004.6:c.71G>A<br>p.Trp24Ter	Pathogenic	2/416	0	0
13:20763677	rs111033217<br>NM_004004.6:c.44A>C<br>p.Lys15Thr	Pathogenic/Likely pathogenic	0	0	-
13:20763686	rs1801002<br>NM_004004.6:c.35G>A<br>p.Gly12Asp	Uncertain significance	0	0	-
	NM_004004.6:c.35G>T<br>p.Gly12Val	Pathogenic/Likely pathogenic	0	0	-
**13:20763686**	**rs80338939**<br>**NM_004004.6:c.35del**<br>**p.Gly12fs**	**Pathogenic**	NA	18/472	0.0089
	**NM_004004.6:c.35dup**<br>**p.Val13fs**	**Pathogenic**	NA	0	-
13:20763687	rs104894408<br>NM_004004.6:c.34G>T<br>p.Gly12Cys	Likely pathogenic	NA	0	0
	NM_004004.6:c.34G>C<br>p.Gly12Arg	Pathogenic	NA	0	-
13:20763702	rs111033451<br>NM_004004.6:c.19C>T<br>p.Gln7Ter	Pathogenic/Likely pathogenic	0	0	-
13:20763710	rs111033222<br>NM_004004.6:c.11G>A<br>p.Gly4Asp	Likely benign	NA	0	0
13:20763712	rs111033401<br>NM_004004.6:c.9G>T<br>p.Trp3Cys	Uncertain significance	0	0	
	NM_004004.6:c.9G>A<br>p.Trp3Ter	Pathogenic/Likely pathogenic	0	0	-
13:20763720	rs111033293<br>NM_004004.6:c.1A>T<br>p.Met1Leu	Likely pathogenic	0	0	-
	NM_004004.6:c.1A>G<br>p.Met1Val	Pathogenic	0	0	-
13:20764940	rs9552099				

	NM_004004.6):c.-22-1198G>T	not reported	1/416	27+3/472	-
	NM_004004.6):c.-22-1198G>A		0	0	0.828
**13:20766921**	**rs80338940**				
	**NM_004004.6:c.-23+1G>A**	**Pathogenic**	0	1/472	-
